# The Use of Bio-Polyurethane Binder for the Development of Engineered Wood Composites

**DOI:** 10.3390/polym17111434

**Published:** 2025-05-22

**Authors:** Sigitas Vėjelis, Agnė Kairytė, Saulius Vaitkus, Arūnas Kremensas

**Affiliations:** Building Materials Institute, Faculty of Civil Engineering, Vilnius Gediminas Technical University, Linkmenu Str. 28, LT-08217 Vilnius, Lithuania; sigitas.vejelis@vilniustech.lt (S.V.); saulius.vaitkus@vilniustech.lt (S.V.); arunas.kremensas@vilniustech.lt (A.K.)

**Keywords:** engineered wood composite, hemp shives, biopolyurethane, mechanical properties, short-term water absorption, reaction to fire

## Abstract

Fiber hemp shives and biopolyurethane binder were used to create an engineered wood composite due to the synergistic properties of these materials. This study created engineered wood specimens using different ratios of biopolyurethane binder and hemp shives, which varied from 0.5 to 1.5. Different pressure levels were used when preparing the specimens, which were 1.5, 3.0, and 4.5 MPa. The formed engineered wood specimens showed that both the amount of binder and the level of pressure significantly influence the strength and moisture indicators, and different processes occur when increasing the amount of binder and the level of pressure. The research showed that the developed engineered wood composites had reached bending strength equal to 17 MPa, tensile strength equal to 7 MPa, and compressive stress equal to 11 MPa. In most cases, the strength index values were higher than those of various industrial-engineered wood products. Engineered wood was characterized by water absorption from 35 to 10%, and swelling in water varied from 26 to 10%. The flammability of the specimens, determined by the low-flame method, indicated that the specimens were flammable, but the expanded graphite used allowed for the creation of non-flammable specimens.

## 1. Introduction

Recycling organic waste is a serious problem in achieving CO_2_ neutrality [1,2]. Two of the simplest and primary methods for the disposal of organic waste can be distinguished: natural degradation of waste in landfills or fields and waste incineration, with or without thermal energy recovery [3,4,5]. In the first case, methane compounds are formed when organic waste degrades naturally, easily entering the atmosphere and contributing to global warming [6,7]. In the second case, during incineration, carbon compounds bound in solid form are released from organic waste and enter the atmosphere in the form of CO_2_ [8,9,10]. Fuels made from plant sources are often not considered a source of CO_2_. Their production does not release more CO_2_ than is absorbed during plant growth; this process is crucial for the greenhouse effect and overall pollution [11,12,13].

Several more effective ways of using organic waste have been proposed in scientific papers. For several decades, wood waste has been successfully used to produce various types of boards—wood fibers [14,15,16], wood chips [17], oriented strand boards [18,19], and different composites [20,21,22,23]. Similar products are also made from straw or processed parts of agricultural plants [24,25,26]. Agricultural residues are an ideal alternative to wood waste because they are abundant, widespread, and readily available. Agricultural waste also has economic, environmental, and technological benefits [27]. The range of alternative materials used for engineered wood composites is extensive and includes rice straw [28,29,30], wheat straw [31,32], bamboo [33], hemp shives [34,35], pineapple leaf fiber [36], castor stalks [37], coconut husks [38], and others. Boards and composites made from organic materials have several disadvantages. First of all, organic fillers are highly flammable. Composites made from these fillers must be designed to improve the fire resistance of the products. Several measures and strategies are employed to produce non-flammable bio-based composites and boards. These include the use of flame-retardant additives [39,40], intumescent systems [41], and surface coatings [42]. A second disadvantage is that many organic fillers absorb much water. Nazmul et al. [43] have determined that hemp shive particles can absorb water up to as much as 335% of their initial weight. Waterproofing agents, coatings, surface treatments, and other techniques increase the water resistance of bio-based composites and boards [44,45,46].

Although large amounts of plant waste are used to produce these boards, the production process is complex, limited in scope, and environmentally unfriendly. The production of such boards requires thermal treatment [47,48,49]. During thermal treatment, not only are considerable energy resources consumed, but the dimensions of the products, especially the thickness, are also limited. During thermal treatment, the beginning and end of the sintering of the mixture in different layers are different, so it is not easy to obtain a homogeneous product. The main drawback of this technology is the use of a formaldehyde-based binder, which requires thermal treatment. Formaldehyde accounts for more than 95% of all adhesives that produce wood-based panels. Such resins are synthesized by reacting formaldehyde with various substances, including urea, phenol, melamine, resorcinol, or combinations [50]. Recently, adhesives derived from fossil resources have increasingly been replaced by bio-based adhesives, e.g., lignin, protein, and tannin-based adhesives [51,52,53]. Each bio-based adhesive type has unique strengths and potential applications, but also several disadvantages. Lignin-based adhesives often exhibit poor water resistance [54], structural heterogeneity, high extracting and purifying costs [55], and relatively low adhesion strength [56]. Protein-based adhesives, such as those derived from fish or soy, destabilize in humid environments [57,58], often exhibit low bonding strength [59], and are prone to mold growth [60]. The limited availability of tannins makes it challenging to source sufficient quantities for large-scale industrial applications [54]. The synthesis of tannin-based adhesives can be costly [61], with low water resistance and overall durability compared to synthetic alternatives [54,61]. In addition to the aforementioned disadvantages, bio-based adhesives usually harden at high temperatures [62,63]. To increase the range of products and the thicknesses of the aforementioned panels, binders that do not require thermal treatment should be used. These binders include polyurethane-based adhesives. The PU industry is still highly dependent on petroleum-based chemicals due to its two primary feedstocks, polyols and isocyanates [64]. PU adhesives are widely used in various industries due to their excellent bonding strength, flexibility, and chemical resistance. They are particularly valued in wood-bonding technologies for creating strong, durable joints [65,66]. Parekh et al. [67] determined that PU adhesives prepared using soybean oil-based polyol and 1,4-butanediol showed the highest bonding strength of 6.36 MPa. PU adhesives can be tailored for specific applications by adjusting the composition and macromolecular architecture, allowing for controlled variations in adhesive strengths and curing times [68]. Recently, increasing attention has been paid to biopolyurethane, in which some of the components obtained from oil are replaced by renewable resources [69,70]. Polyurethanes are formed by reacting with di, tri, or polyisocyanate groups and polyols, subjected to an exothermic reaction [71]. This formation occurs in the presence of a chain extender, catalyst, and/or additives such as ester, ether, urea, and aromatic rings existing in the backbone of polyurethane [72].

Bio-based polymers have numerous benefits, such as abundant availability, low price, and environmental benignity. Vegetable oils are commonly converted into derivatives, such as alkyd resins and alkyd-based polyols. Polyols are then reacted with different diisocyanates to obtain PU coatings. Bio-based polyurethanes may be synthesized from vegetable oils from plant seeds such as neem, castor, cotton, rapeseed, jatropha, palm, and soybean, etc. [73]. The development of bio-based polyurethanes has shown promise in reducing reliance on petrochemical resources and improving environmental sustainability [70,74,75].

This study aims to create engineered wood products from hemp shives and biopolyurethane without thermal treatment. The creation of engineered wood without thermal treatment allows for the production of products of unlimited dimensions, which, in many cases, allows natural wood to be replaced in producing boards, rafters, logs, and the like.

## 2. Materials and Methods

### 2.1. Raw Materials

Hemp shives obtained from the processing of the hemp stalks were used as fillers in the production of engineered wood. Hemp shives were taken from “Natūralus pluoštas” (Kėdainių District, Lithuania). The shives of the hemp variety “Austa SK” were used. A fraction of 0–20 mm fibrous hemp shives was obtained.

The ratio of biopolyurethane binder to hemp shives varied from 0.5 to 1.5 in steps of 0.25. The biopolyurethane binder consisted of isocyanate, polyol, and natural rapeseed oil. Polymeric 4,4-diphenylmethane diisocyanate Lupranat M20S with 31.5% NCO (BASF, Berlin, Germany) was used as a hardener. Polyol Biopolyol RD was produced in SIA PolyLabs (Riga, Latvia). Rapeseed oil-based polyol had a hydroxyl number of 350 KOH/g and less than 0.2% water. Rapeseed oil was produced by Lomista UAB (Kaišiadorys, Lithuania). Expandable graphite was used to reduce the flammability of the engineered wood. The expandable graphite ES 350 F5 was manufactured by Graphit Kropfmühl GmbH (Hauzenberg, Germany). The main parameters of the expandable graphite were as follows: particle size: < 71 µm 98.0%, carbon content: 99.70%, expansion rate: 350–700 cm^3^/g, and starting temperature: 180–240 °C.

Table 1 gives the compositions of the mixtures obtained from the prepared raw materials. The amounts of binding material and expandable graphite were calculated based on the mass of hemp shives; therefore, the amount of hemp shives was taken as 100%.

### 2.2. Preparation of Binding Material, Mixture, and Specimens

**Figure 1:**
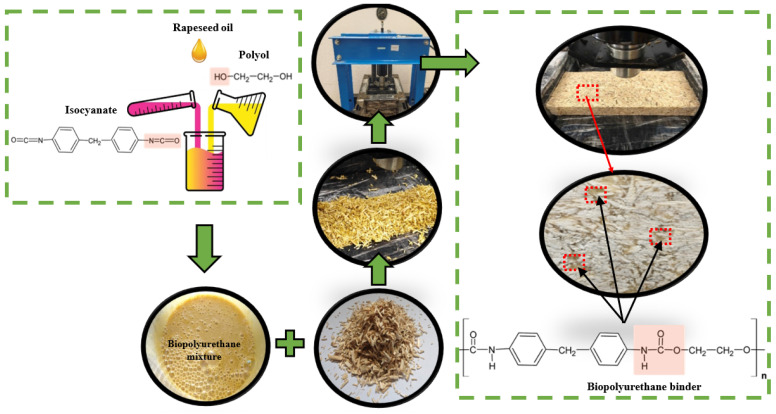
The entire specimen-preparation process.

First, polyol and rapeseed oil were mixed for a minute at 1800 rpm. After that, the intended amount of isocyanate was added to the binding material, produced, and stirred for another 30 s. The percentage ratio of the binder composition was as follows: isocyanate—35%, polyol—44%, and rapeseed oil—21%. The prepared biopolyurethane binder was poured onto the weighted shive particles and thoroughly mixed for a minute at 1800 rpm. When expandable graphite is used to prepare the engineered wood composite, the required amount is evenly spread on the hemp shives and the hemp shives are additionally mixed for one minute at 1800 rpm with the expandable graphite, before mixing them with the prepared biopolyurethane binder. The mixture was evenly poured into a metal mold covered with polyethylene film to prevent polyurethane from sticking to the mold, pressed with a required load using a pneumatic–hydraulic press Tongrun T40 (Shanghai Tongrun Imp. & Exp. Co., Ltd., Shanghai, China), and it was kept compressed for 20 min before demolding and cutting of the hemp shive-based engineered wood specimens (further in the text, engineered wood) to the required dimensions took place. The dimensions of the prepared prototype specimen in the mold were 320 × 100 × (30 ÷ 50) mm. The height of the specimen varied, depending on the pressure level and the amount of binder. The entire specimen-preparation process and binding-material reactions are illustrated in Figure 1.

### 2.3. Characterization Methods

Before preparing the specimens, the original edges and surfaces of the prototype were removed to avoid unevenness. The specimens from the prototypes were prepared so that they were tested perpendicular to the direction of the formation of the prototype.

The strength characteristics of the engineered wood were determined using a computerized press HOUNSFIELD H10KS (Hounsfield Ltd., Salfords, UK), and the computer software QMAT PROFESSIONAL v. 2.10 was adapted to it. The prepared specimens’ bending strength and modulus of elasticity were determined according to the methodology specified in the EN 310 standard [76]. Bending tests were performed by applying a load to the center of the specimen, which was placed on two supports. Three specimens of each composition were prepared for bending tests, each measuring 290 × 50 × 12 mm, to ensure the standard requirement for a specimen length of (20 × t + 50) × 50 × t mm, where t is the specimen thickness. The specimens prepared for the bending test were conditioned for 72 h at a temperature of 23 ± 2 °C and 50 ± 5% relative humidity.

The specimens’ tensile strength and modulus of elasticity perpendicular to the board plane were determined based on the methodology specified in the EN 319 standard [77]. The tests were performed using self-aligning grips with ball joints to clamp the specimen. Three specimens of each composition with dimensions of 20 × 20 × 20 mm were prepared for this test. The glued set of plates and the specimen were kept for 72 h at a temperature of 23 ± 2 °C and 50 ± 5% relative humidity. After the specified holding time, the specimen set was placed in the grips and subjected to force until it disintegrated.

To determine the compressive stress and modulus of elasticity in compression, three specimens of size 30 × 30 × 30 mm of each composition were prepared. The compression parameters were determined according to the ISO 29469 standard methodology [78]. The specimens prepared for the compression tests were conditioned for 72 h at a temperature of 23 ± 2 °C and 50 ± 5% relative humidity.

The short-term water absorption of specimens prepared from flat prototypes was determined by immersing the specimens in water for 24 h, according to the requirements of EN 1609 standard method A [79]. After the specified time, the specimens were removed from the water, and the excess water not absorbed was allowed to drain. For the test, three specimens with dimensions of 50 × 50 × t mm were prepared from each series of composites. Before the start of the test, the specimens were conditioned for at least 6 h at 23 ± 5 °C. Water at a temperature of 23 ± 5 °C was poured into the bath with the specimens until its level above the lower plane of the specimen reached a level of 10 ± 2 mm. The immersed specimens were left in water for 24 ± 0.5 h.

Thickness swelling tests of the prepared specimens were performed according to EN 317 [80]. Thickness swelling was determined by measuring the specimen’s thickness change after being stored in water. Before the start of the test, the specimens were conditioned for 72 h in an environment with a temperature of 23 ± 5 °C. For the test, three specimens with dimensions of 50 × 50 × t mm were prepared from each series of composites.

Combustibility tests of manufactured engineered wood composites were conducted according to the methodologies specified in the EN ISO 11925–2:2010 [81] standard. This test determines the propagation of a small flame up the vertical surface of the specimen when the surface or edge of the specimen is exposed to a small flame for an appropriate period. Three specimens were prepared from each composition, with a length of 250 ± 2 mm and a width of 90 ± 2 mm. Before the test, the specimens were conditioned in an environment with a temperature of 23 ± 2 °C and 50 ± 5% relative humidity for 72 h. The specimens were exposed to the flame for 15 s. The flame was directed towards the specimen at a 45° angle and at the correct distance from the burner. The burner valve adjusted the flame height to (20 ± 1) mm. After adjusting the parameters, the burner moved horizontally until the flame reached the pre-set contact point with the testing specimen. When the flame touched the specimen, the time device was triggered, and, after 15 s, the burner was eliminated. The test specimen was left in the combustion chamber for another 120 s. In the case of specimen burning, the process was terminated. After the set duration of flame operation, the test data were recorded. These comprised whether the specimen ignited; whether the top of the flame reached a height of 150 mm above the point of flame action and after what time; whether there were falling flaming drops or particles that ignited the filter paper, whether the specimen smoldered and for how long; and other observations regarding physical changes in the specimen.

The effect of flame retardants on the engineered wood specimens’ flammability was evaluated using a scanning electron microscope JEOL JSM-7600F (JEOL, Tokyo, Japan).

Thermogravimetric (TGA) and differential thermogravimetric (DTG) analyses were conducted with the TG 209 F1 analyzer (Netzsch Group, Selb, Germany) in the temperature range from 25 °C to 800 °C, with a temperature rising speed of 10 °C/min, and with an air atmosphere.

## 3. Results and Discussion

### 3.1. Evaluation of Mechanical Properties

**Figure 2:**
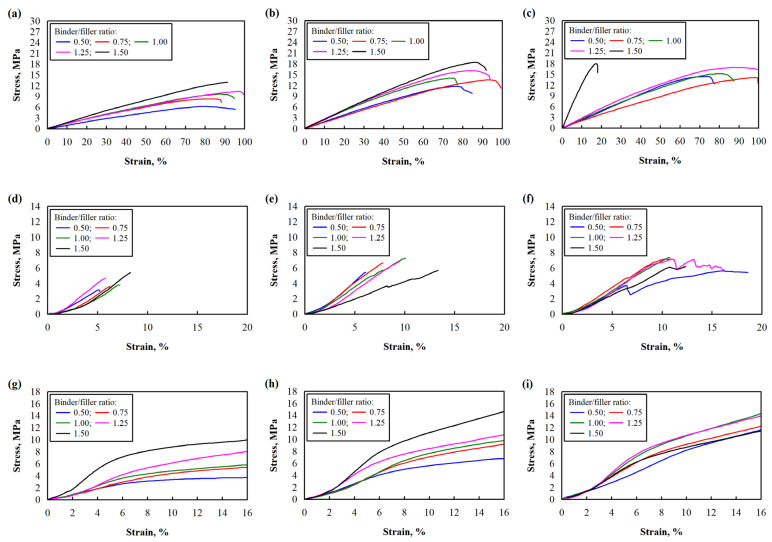
The stress–strain curves of the mechanical tests: (**a**) bending of specimens with mixture pressure 1.5 MPa; (**b**) bending of specimens with mixture pressure 3.0 MPa; (**c**) bending of specimens with mixture pressure 4.5 MPa; (**d**) tensile of specimens with mixture pressure 1.5 MPa; (**e**) tensile of specimens with mixture pressure 3.0 MPa; (**f**) tensile of specimens with mixture pressure 4.5 MPa; (**g**) compression, specimens with mixture pressure 1.5 MPa; (**h**) compression, specimens with mixture pressure 3.0 MPa; (**i**) compression, specimens with mixture pressure 4.5 MPa.

The strength and modulus of elasticity in bending, tensile, and compression were evaluated during the tests. The curves of the mechanical tests are shown in Figure 2.

**Figure 3:**
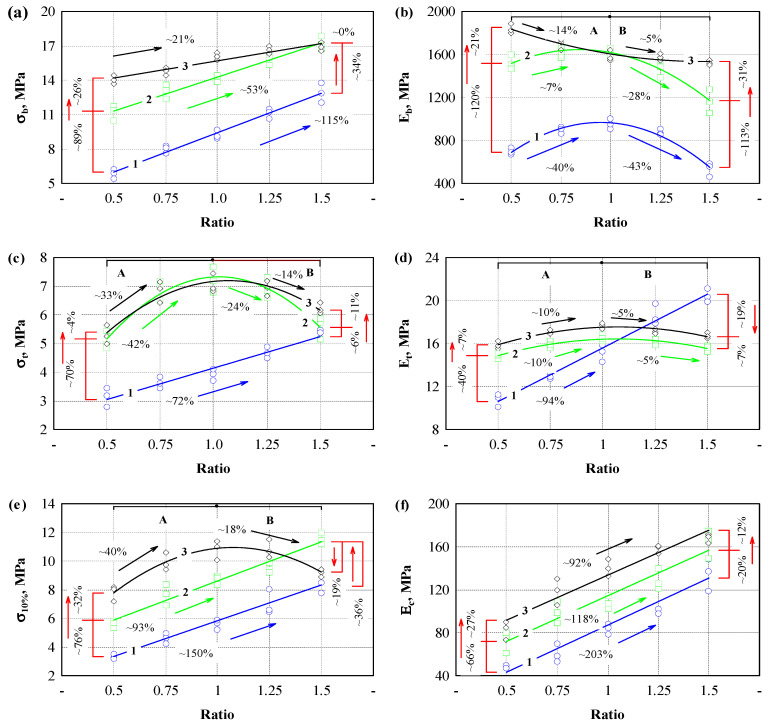

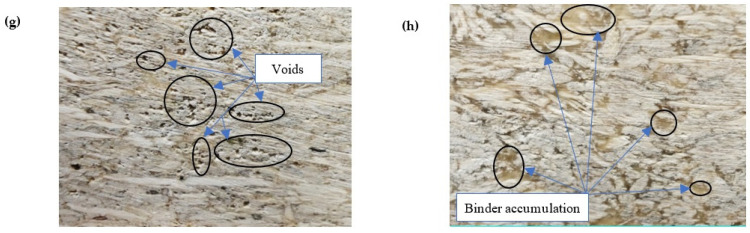
Dependence of the strength parameters on the ratio of binding material and aggregate and the pressure level: (**a**) bending strength; (**b**) tensile strength; (**c**) compressive stress at 10% deformation; (**d**) bending modulus of elasticity; (**e**) tensile modulus of elasticity; (**f**) compression modulus of elasticity; (**g**) pressure level 4.5 MPa and binder/filler ratio 0.5; (**h**) pressure level 4.5 MPa and binder/filler ratio 1.5. A—is the zone when the parameter increases; B—is the zone when the parameter decreases. 1 (blue line)—pressure level 1.5 MPa; 2 (green line)—pressure level 3.0 MPa; 3 (black line)—pressure level 4.5 MPa. Black, green, blue and red arrows—increment or reduction of the parameter.

The presented curves show differences between samples prepared with different binder/filler ratios and pressures. The obtained test values were graphically represented in Figure 3 to evaluate these differences. Figure 3a shows the dependence of bending strength on the binder/filler ratio when the specimens were prepared with 1.5 (Figure 3a, line 1), 3.0 (Figure 3a, line 2), and 4.5 (Figure 3a, line 3) MPa of pressure. In all cases, we can observe that, with increasing binder content, the values of bending strength also increase. The intensity of the increase in strength values differs for every pressure level and binder/filler ratio. The greatest intensity of the increase in bending strength values when the binder/filler ratio is increased from 0.5 to 1.5 was observed when 1.5 MPa pressure was used. In this case, this increase reached 115%. When using 3.0 MPa pressure, a 53% increase in strength values was observed, and when using 4.5 MPa pressure, a 21% increase was observed. The influence of pressure level on the bending strength was evaluated separately. The lowest bending strength was observed in specimens formed using 1.5 MPa pressure. Their bending strength ranged from 7 to 12.5 MPa. When comparing specimens formed with 3.0 and 4.5 MPa pressures, the most significant difference of 26% was observed when the smallest amount of binder was used, and when the highest amount of binder was used, there was no difference.

Different trends were observed when evaluating the elastic modulus values (Figure 3b). In the case of bending strength, linear dependences on the number of binder/fillers were observed. Curves separated into zones A and B were observed in the case of the bending elastic modulus. An increase in the elastic modulus values was observed when using a pressure of 1.5 MPa and when increasing the binder/filler ratio to 1. A decrease in the modulus values was observed when using a binder/filler ratio of 1.25 and 1.5. When the binder/filler ratio changed from 0.5 to 1, the volume of the binding material was small and filled the air spaces between the hemp shives. Therefore, the bending process occurred through the biopolyurethane and the shives framework. When the amount of binder was increased, then it is likely that a continuous biopolyurethane network was formed, which is more easily deformed under load than with hemp shives. Similar trends in the elastic modulus were also observed in the specimens for the formation of which a pressure of 3.0 MPa was used, with significantly higher modulus values ranging from 113 to 120%.

Meanwhile, when forming the specimens using a pressure of 4.5 MPa, the bending modulus curve changes substantially. The highest flexural modulus was obtained with a small binder but high pressure. Increasing the amount of binder decreased the bending modulus until a binder/filler ratio of 1:1 was reached. The elastic modulus values remained similar with a further increase in the amount of binder. The binder likely filled all the pores due to the high pressure, and then its amount between the hemp shives increased (Figure 3g).

The tensile strength results are presented in Figure 3c. The test shows the specimen’s strength values and the effectiveness of the binder–filler adhesion. The tensile strength increased intensively by using a constant pressure of 1.5 MPa and increasing the binder content (Figure 3c, zone A). Increasing the binder content three times increased the tensile strength by 72%. The strength values also increased when testing the composite with the lowest binder content, but at an increasing pressure. The most significant increase in tensile strength values was observed using pressures of 3.0 and 4.5 MPa. In this case, a 70% increase in tensile strength was obtained, i.e., from 3.1 MPa to 5.2 MPa. An intensive increase was also observed when the binder/filler ratio was increased to 0.75 and 1. In this case, an 86% and 73% increase in tensile strength was observed, respectively. However, when the binder/filler ratio was increased to 1.25, a slight decrease in strength values was observed, and when the binder/filler ratio was increased to 1.5, a sharp decrease in tensile strength began (Figure 3c, zone B). When using a pressure of 4.5 MPa, the strength values in all sections were very close to those under a pressure of 3.0 MPa.

All of the formed engineered wood composites’ tensile modulus of elasticity increased until a binder/filler ratio of 1 was reached (Figure 3d, zone A). By further increasing this ratio, the elastic modulus of the specimens prepared at different pressures changed differently. The elastic values of the specimens formed with a pressure of 1.5 MPa continued to increase and, at a ratio of 1.5, exceeded the elastic modulus values of the specimens formed with pressures of 3.0 and 4.5 MPa. Meanwhile, the elastic modulus values of the specimens formed with a pressure of 3.0 and 4.5 MPa remained almost constant (Figure 3d, zone B).

Figure 3e graphically depicts the compressive stress results at 10% deformation. The lowest strength was observed for the specimens of engineered wood formed with a binder/filler ratio of 0.5 and using a compressive stress of 1.5 MPa (Figure 3e, zone A). When the binder/filler ratio was increased to 1.5, the compressive stress increased by 72%. Increasing the pressure level to 3.0 MPa increased the compressive strength from 93% to 117% compared to the specimens with a pressure level of 1.5 MPa.

When a pressure of 4.5 MPa was used and the binder/filler ratio was from 0.5 to 1, the compressive strength increased by more than 30% compared to the specimens formed with a pressure of 3.0 MPa. A decrease in the compressive strength values was observed with a further increase in the binder/filler ratio (Figure 3e, zone B).

The compressive modulus of elasticity results are presented in Figure 3f. Increasing the binder/filler ratio from 0.5 to 1.5 and increasing the compressive stress from 1.5 to 4.5 MPa led to a consistent increase in the elastic modulus values. If increasing the amount of binder/filler leads to a uniform increase in the values of the compressive modulus, then increasing the pressure level from 1.5 to 3.0 MPa results in an approximately twice greater increase in the values of the compressive modulus than increasing the pressure level from 3.0 to 4.5 MPa. It means that the influence of the pressure level on the values of the elastic modulus begins to have less significance.

When summarizing the results of the bending, tensile and compressive strengths, and elastic modulus, the optimal binder/filler ratio is 1. A larger amount of binder leads to the formation of weak zones and the deterioration of individual strength and deformation characteristics. In addition, it was observed that the molding mass was extruded on the sides of the specimens in some cases during the formation of the specimens. When examining all specimens, it was found that the molding mass was not extruded when using 1.5 MPa pressure and any binder/filler ratio from 0.5 to 1.5. When using 3.0 MPa pressure and binder/filler ratios of 1.25 and 1.5, extruded mass formations formed on the surface of the specimens. When using 4.5 MPa pressure, the appearance of the extruded mass was observed when using a binder/filler ratio of one or more. It was found that the mass was extruded immediately after the specimen was pressed, and this process lasted for another 2–3 min. In addition, when analyzing the extruded mass, it was found that only biopolyurethane binders without hemp shives were extruded because, under load, they easily move between the shives. When such a load is reached, the shives begin to compact, and the biopolyurethane binder begins to push through the shives outward.

When evaluating the pressure level used for forming engineered wood, the pressure of 1.5 MPa was too low because small voids remain in the composite structure, which determines the lower values of strength indicators. Meanwhile, when using a pressure level of 3.0 MPa and a binder/filler ratio equal to 1, the strength characteristics were close to those of a pressure level of 4.5 MPa. However, no extruded mass formations were observed at the edges of the specimens.

Empirical equations describe the strength and modulus of elasticity in bending, tension, and compression (see Table 2). Equations are provided for each pressure level.

**Figure 4:**
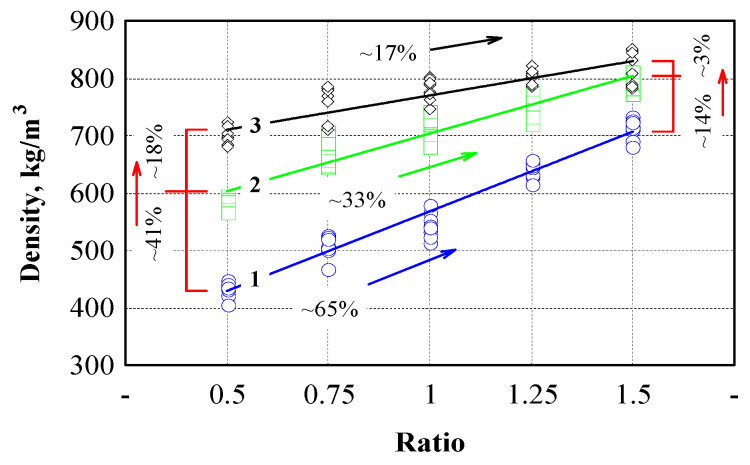
Dependence of the density of engineered wood composites on the binder to hemp shives ratio. 1 (blue line)—pressure level 1.5 MPa; 2 (green line)—pressure level 3.0 MPa; 3 (black line)—pressure level 4.5 MPa. Black, green, blue and red arrows—increment or reduction of the parameter.

The influence of binder and pressure level on the density of engineered wood was evaluated (Figure 4, Equations (19)–(21) in Table 3). The lowest density was observed in composites with the lowest binder content. When the binder content was increased, the density increased intensively, but the pressure level also significantly influenced the intensity of the density increase. If the lowest density was observed in engineered wood formed with a binder/filler ratio of 0.5 and a pressure of 1.5 MPa, then, at the same pressure using a binder/filler ratio of 1.5, the density increased by 65%, while, when using a pressure of 3.0 MPa, a 33% increase was observed, and, when using a pressure of 4.5 MPa, only a 17% increase in density was observed. A decrease in the intensity of density growth was also observed when the pressure level increased. When forming engineered wood specimens with a binder/filler ratio of 0.5, the density increase reached 41% when the pressure was increased from 1.5 to 3.0 MPa and only 18% when the pressure was increased from 3.0 to 4.5 MPa.

When using a binder/filler ratio of 1.5 and the pressure being increased from 1.5 to 3.0 MPa, the density increase reached 14%, and only 3% when the pressure was increased from 3.0 to 4.5 MPa. Such a decrease in the intensity of density growth can be explained by the different compaction processes of hemp shives, which are shown in Figure 2. The production of composites is given much attention in scientific works. Raw materials, technology, and the purpose of composites vary greatly. In particleboard production, pressure is one of the main indicators determining the product’s properties [82]. The pressure level in the wood particleboard industry is usually specified in the range of 3.0 to 3.5 MPa [83].

Typically, the product’s density, thickness, and mechanical strength depend on the compression level [84,85]. In our case, the selection of pressure was more related to the amount of binder used. Formaldehyde-based adhesives, particularly urea-formaldehyde, are widely used in particleboard production with typical proportions ranging from 6% to 12 [86]. Our research has shown that the best properties are achieved when 50% of the binder is used. Various types of cement are often used as a binder for composites without thermal treatment. The authors of [87] used corn stalks as a filler and magnesium phosphate cement-based binder. The amount of corn stalk in the composite varied from 5 to 30%, and the density from 557 to 1854 kg/m^3^. In our case, the density of the composites varied from 420 to 820 kg/m^3^, and the compressive stress from 3.5 to 11.0 MPa.

Meanwhile, in one study [88], the compressive strength of the composites created with a cement binder in this density range varied from 2.0 to 4.5 MPa. According to EN 312 [89] standard requirements for the particleboards for use in dry conditions, when the nominal thickness of boards ranges from ˃6 to 13 mm, the lowest bending strength must be 10.5 MPa and the tensile strength must be 0.28 MPa. In our research, the highest bending strength was 17 MPa, and the tensile strength was 7 MPa. For broader use of our engineered wood composite, increasing the elastic modulus values is necessary.

### 3.2. Evaluation of Water Impact

**Figure 5:**
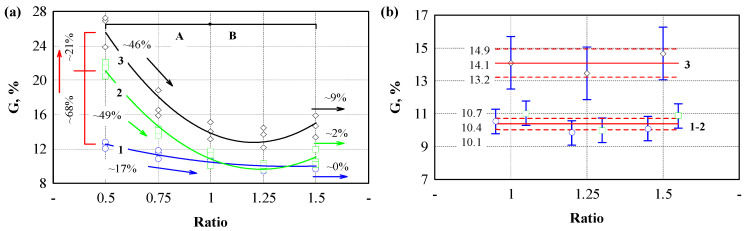
Dependence of engineered wood’s swelling on the binder and hemp shives ratio. (**a**) swelling kinetics at different ratios of binder and hemp shives; (**b**) swelling analysis in the steady state zone; A—is the zone when the parameter decreases; B—is the zone when the parameterdoes not change. 1 (blue line)—pressure level 1.5 MPa; 2 (green line)—pressure level 3.0 MPa; 3 (black line)—pressure level 4.5 MPa. Black, green, blue and red arrows—direction of change of the parameter.

The results of the swelling of engineered wood specimens in water are presented in Figure 5 and Table 4. The results of the studies show that the highest swelling in all cases was observed in specimens formed with the lowest amount of biopolyurethane. The swelling intensity also significantly depends on the pressure level used. When a compressive stress of 4.5 MPa and a binder/filler ratio of 0.5 were used, the swelling value reached 26%. When the pressure level of the specimens was reduced to 1.5 MPa, the swelling values were reduced by half, i.e., to 13%, and when using a pressure of 3.0 MPa, to 21%. A sharp drop in swelling values was observed when the binder/filler ratio reached one. Moreover, when using a pressure level of 4.5 MPa for specimens, the swelling value was 14%, and when using pressure levels of 3.0 and 1.5 MPa, the swelling values were only 11% and 10.5%, respectively.

With further binder/filler ratio increase, swelling values remained almost constant. Statistical analysis of the results shows (see Figure 5b) that when using a specimen pressure level of 4.5 MPa, swelling values obtained when the binder/filler ratio was varied from 1 to 1.5 did not differ from values obtained when using 1.5 and 3.0 MPa pressure. In this section, swelling values obtained when using specimen pressures of 1.5 and 3.0 MPa did not differ among themselves or when changing the binder/filler ratio. It can be assumed that, when reaching a binder/filler ratio equal to one, biopolyurethane completely covers the surface of hemp shives and acts as a water-insulating barrier.

Meanwhile, when using a higher pressure level but an insufficient amount of binder, a strong capillary suction effect was observed, which was manifested by high swelling values. The walls of hemp shive cells were likely strong enough to withstand the stresses that occur during swelling. Since hemp cell walls are partially compressed under high compressive stresses, i.e., 4.5 and 3.0 MPa, during swelling by capillary suction, the walls of hemp cells swell again to the maximum wall stress. With an increase in the amount of binder, the swelling of the walls was limited, primarily due to the reduced voids and, secondarily, due to the sufficient strength of the cured biopolyurethane.

**Figure 6:**
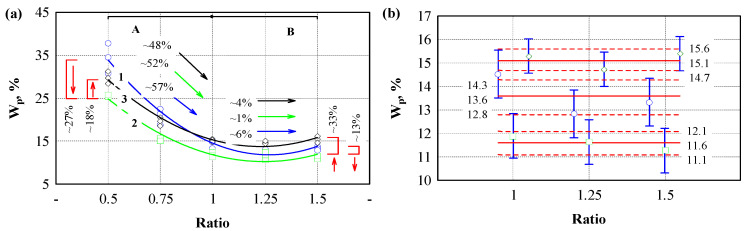
The dependence of engineered wood water absorption on the binder and hemp shives ratio. (**a**) water absorption kinetics at different ratios of binder and hemp shives; (**b**) water absorption analysis in the steady state zone. A—is the zone when the parameter decreases; B—is the zone when the parameter does not changes. 1 (blue line)—pressure level 1.5 MPa; 2 (green line) –pressure level 3.0 MPa; 3 (black line)—pressure level 4.5 MPa. Black, green, blue and red arrows—direction of change of the parameter.

The results of the short-term water absorption of engineered wood specimens are presented in Figure 6. The statistical processing results are presented in Table 5.

The trends of water absorption are similar to those of swelling, but there are some nuances that differ. Water absorption values with the lowest binder content reached 35%, and the lowest water absorption was observed when the filler/binder ratio reached one and decreased. The results of the studies show that the amount of binder used has the most significant influence on the results.

Other researchers [90] have stated that hemp particleboards showed higher water absorption and swelling levels than wood-based particleboards. The authors determined that the average swelling of the hemp particleboard was 8.97%, and its average water absorption was 480.11%. The water absorption of specimens made with cement binder [88] was very closely related to the amount of filler used, with 5% filler water absorption not reaching 2%, and, with 30% filler, it reached 50%. In another work [91], pine wood waste, sugarcane bagasse, and polyurethane or urea formaldehyde were used as adhesives in particleboards. The binder content was only 10%. In these specimens, water absorption varied from 10.5 to 31.0%, and swelling varied from 6.04 to 18.06%. These results are similar to those obtained in our study. Since hemp fibers tend to absorb much water, and their content in an engineered wood composite can be more than 50 percent, treating hemp fibers with various coatings or hydrophobicizers can help reduce the impact of water on the entire composite several times over. The selection of coatings or hydrophobicizers should not impair the formation of contact zones between the hemp fibers and the biopolyurethane binder.

### 3.3. Evaluation of the Influence of Expandable Graphite on Strength and Water-Absorption Characteristics

**Figure 7:**
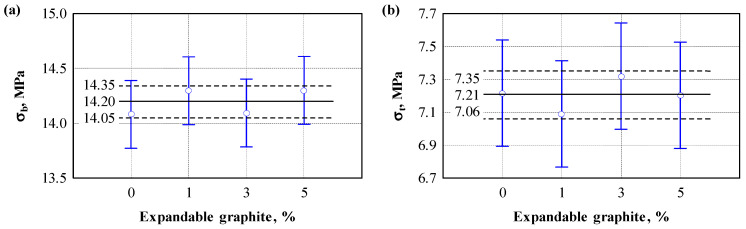

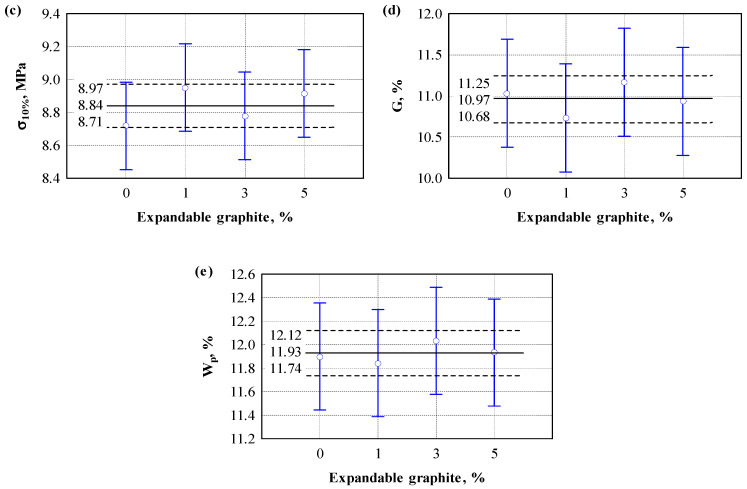
The influence of expandable graphite on the strength and water-absorption characteristics of engineered wood composite: (**a**) bending strength; (**b**) tensile strength; (**c**) compressive stress; (**d**) swelling; (**e**) water absorption.

During the tests, the influence of expandable graphite on strength and water absorption characteristics was evaluated. Figure 7 shows a statistical analysis of the test results.

When performing a one-way analysis of variance (Table 6) of the physical properties, the aim was to conclude whether there was a statistically significant difference between the test groups (e.g., bending strength 0%, 1%, 3%, 5%). The results obtained showed that the means of the (bending, tensile, compression, swelling and absorption) groups did not statistically show a significant difference (F(3,8) < Fcr, *p* > 0.05).

Based on the obtained data on physical properties, the null hypothesis of rejection was not established, which allows us to state that the differences in the mean values between the test groups (bending strength, tensile strength, compressive stress, swelling, and water absorption) could have occurred by chance. Therefore, there is no sufficient reason to believe that the mean of at least one group differs significantly from the other.

It can be assumed that a small amount of expanded graphite does not affect the strength and water absorption indicators of engineered wood composites or that this influence is insignificant.

### 3.4. Assessment of Ignitability

**Figure 8:**
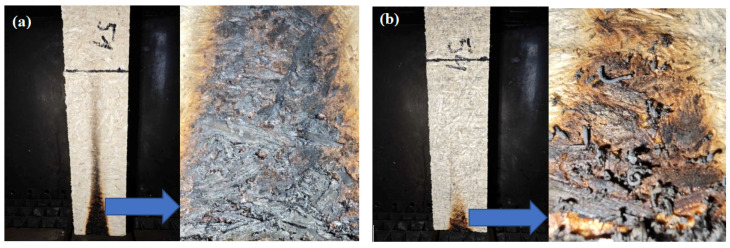
View of the specimens after flammability tests: (**a**)—specimen without flame retardant; (**b**)—specimen with expandable graphite.

Figure 8a shows an image of an engineered wood specimen after combustion without flame retardant. The photo shows that the charring of the specimen surface, due to the flame’s effect, covers a considerable height of the specimen, and a carbon layer is formed on the surface exposed to the fire.

**Figure 9:**
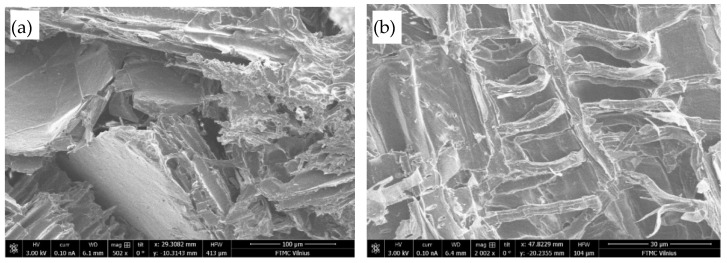
View of engineered wood composite structure with flame retardant after flammability tests at magnifications of (**a**) ×500; (**b**) ×2000.

Figure 8b shows an image of the structure with flame retardant. The photo shows that the flame affects only the area where the flame source is located. A carbon layer of uneven structure is formed on the specimen’s surface exposed to the fire. This carbon layer was examined using scanning electron microscopy (see Figure 9).

In Figure 9, the expansion of graphite particles is observed in the structure of the specimen—a whole series of small ribbons are formed, with a diameter of is 2–4 μm, a length of 40–90 μm, and the gaps between individual ribbons being 5–10 μm. It is likely that several rows of such ribbons form during combustion, and through their tangle, air reaches the material’s surface with great difficulty and very slowly, so the flame goes out due to the lack of air.

**Figure 10:**
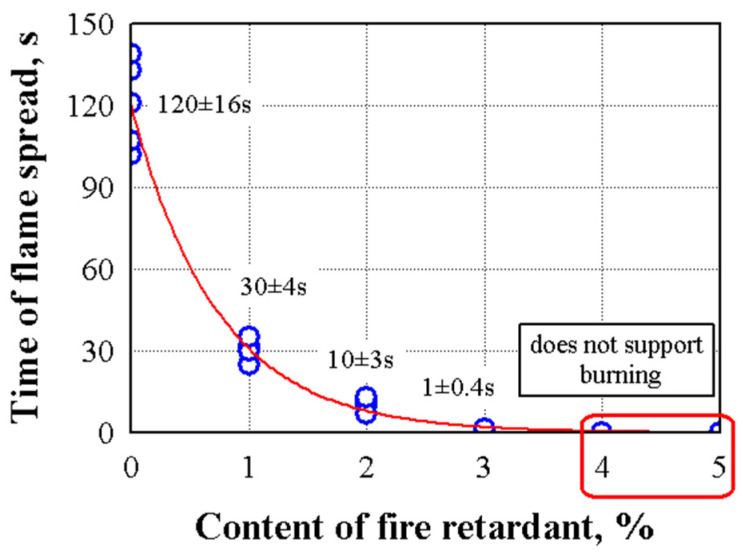
The dependence of flame spread depends on the amount of flame retardant (the amount of flame retardant is calculated per 1 kg of dry shives).

Figure 10 shows the impact of flame-retardant additives on the flame spread of specimens without expanded graphite and 1 to 5% graphite. When no flame retardant was used, the flame reached the 150 mm mark within 120 s. After removing the flame source, the flame slowly subsided and went out after about 120 s. In the specimens with flame retardant, depending on the amount of flame retardant, the flame did not spread or spread very little on the surface of the specimen. Studies show that a 4% flame-retardant content by mass of the material completely stops the spread of fire. Other authors [92] note that hemp shives and sapropel composites are flammable. However, their flammability can be regulated using fire-smothering liquids, fire retardants, and the formation of different protective coatings.

Flammability tests have shown that the addition of expanded graphite, depending on its amount, can effectively suppress flammability. We see that, when 1% of the additive of expanded graphite was added to the specimen, the flame propagation rate decreased from 120 to 30 s, that is, 4 times, and when 3% of the additive of expanded graphite was added, the flame propagation rate decreased from 120 to 1 s, that is 120 times. When the amount of expanded graphite was increased from 4 to 5%, the specimen did not support flammability.

The statistical analysis has shown that the experimental results of the flammability tests can be described by the following Equation (28):

(28) $$Time\;of\;flame\;spread=120.333\left(exp\left(-Graphite\cdot 1.35624\right)\right),$$

with standard deviation $S_{r}=6.1$ s (n = 30) and the coefficient of determination $R^{2}=0.981$. The value of the coefficient of determination shows that changes in the flame spread rate are influenced by an average of 98.1% by the change in the amount of expanded graphite and only 1.9% by other factors.

**Figure 11:**
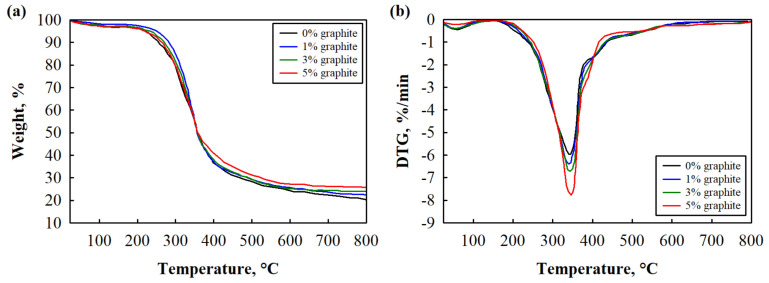
Thermal degradation curves of engineered wood composites at binder/filler ratio 1.00: (**a**) TGA and (**b**) DTG.

The thermograms of the analyzed engineered wood composite samples (Figure 11) were used to determine the temperatures at 5% and 50% weight loss (T_5wt.%_ and T_50wt.%_), the maximum degradation rate (T_max_), and char yield at 800 °C. These parameters are summarized and presented in Table 7.

Even though the DTG curves show one visible peak of T_max_ around 340 °C, the thermal decomposition of engineered wood composites is complex and can be divided into three decomposition stages. The first one is a small band up to 105 °C. This is apparent due to the moisture in the samples and the release of residual volatile substances [93]. The subsequent thermal degradation stage around 300–400 °C is assigned to the cellulose in hemp shives, along with the degradation of soft segments and the decomposition of hard segments in the biopolyurethane binder. The last stage of thermal degradation occurs around 400–500 °C. According to Olszewski et al. [94], it can be associated with the breakdown of lignin in hemp shives and the longest chains within the soft segments of biopolyurethane binder.

Quantitative analysis of the results in Table 7 is surprising in the case of all engineered wood composites with the 1% and 3% addition of expandable graphite, indicating enhanced initial thermal stability. It is visible mainly during the comparison of T_5wt.%_, which shows that the degradation temperature increased from 219 °C to 247 °C and 231 °C, respectively, compared to the control engineered wood composite without expandable graphite. However, the further addition of expandable graphite to 5% resulted in T_5wt.%_, similar to the control engineered wood composite sample. As Shafigullin et al. [95] determined, up to 4% expandable graphite forms a denser intumescent layer, which causes slower diffusion of volatile combustible fragments, and further addition of expandable graphite reduces the thermal stability of the resulting engineered wood composites. It can be assumed that more than 5 wt.% expandable graphite possibly inhibits the effective intumescent layer formation.

Further, no significant changes can be seen in T_max_, and T_50wt.%_ is identical for all of the analyzed engineered wood composite samples. Notably, the char yield at 800 °C correlates with expandable graphite concentration, indicating that expandable graphite contributes to increased residual mass at higher temperatures.

## 4. Conclusions

The strength indicators of engineered wood are greatly influenced by the amount of binder and the mixture’s pressure level. Too low a pressure level or amount of binder does not ensure the formation of reliable contact zones. Too high a pressure level or amount of binder leads to the release of the binder from the mixture and uneven distribution of the binder. It has been determined that the optimal binder–filler ratio is 1:1, and the mixture’s pressure level is 3.0 MPa.

The lowest density of 430 kg/m^3^ was observed in engineered wood when the mixture’s lowest pressure level was 1.5 MPa, and the lowest binder–filler ratio was 0.5. Such a density does not ensure the best properties of engineered wood. The rational density of engineered wood is 700 kg/m^3^, which ensures the best strength indicators.

The lowest swelling was observed in engineered wood specimens prepared using the lowest mixture pressure of 1.5 MPa and a binder–filler ratio of 1.0 to 1.5. Similar trends were observed when determining the impregnation results. The mixture’s pressure level shows that the higher the amount of hemp shives in a given volume of material, the higher the impregnation. This means that hemp shives have the most significant influence. This is also confirmed by the influence of the binder amount on the swelling and impregnation results. When the binder amount is higher, the hemp shives are better insulated from the effects of water, and then the swelling and impregnation values decrease.

Flammability tests have shown that engineered wood is flammable. However, when the appropriate amount of 4% expanded graphite is used, engineered wood becomes non-flammable—the flame does not spread, and combustion does not occur after the fire source is removed.

## Figures and Tables

**Table 1:** Compositions of boards forming mixtures.

Mixture No.	Hemp Shives, %	Polyurethane Binder, %	Expandable Graphite, %
1-1	100	50	0
1-2		75	
1-3		100	
1-4		125	
1-5		150	
2-1		100	1
2-2			2
2-3			3
2-4			4
2-5			5

**Table 2:** Statistical analysis of the processing of engineered wood strength.

Figure	Specimen	Statistical Characteristics								
		$b_{0}$	$b_{1}$	$b_{2}$	R	R^2^	Adjusted R^2^	$S_{r}$	F	*p*
Bending strength										
3a	15	$\mathrm{Level}\;\mathrm{of}\;\mathrm{pressure}\;(1\;*)\text{:}\;\sigma_{b}=b_{0}+b_{1}\cdot Ratio$ (1)								
		2.54867	6.87867		0.983	0.967	0.964	0.483	380.4	0.0
	15	$\mathrm{Level}\;\mathrm{of}\;\mathrm{pressure}\;(2\;*)\text{:}\;\sigma_{b}=b_{0}+b_{1}\cdot Ratio$ (2)								
		8.3360	5.94267		0.977	0.955	0.951	0.490	275.3	0.0
	15	$\mathrm{Level}\;\mathrm{of}\;\mathrm{pressure}\;(3\;*)\text{:}\;\sigma_{b}=b_{0}+b_{1}\cdot Ratio$ (3)								
		12.68867	3.00800		0.942	0.887	0.879	0.407	102.3	0.0
Bending modulus of elasticity										
3b	15	$\mathrm{Level}\;\mathrm{of}\;\mathrm{pressure}\;(1\;*)\text{:}\;E_{b}=b_{0}+b_{1}\cdot Ratio+b_{3}\cdot Ratio^{2}$ (4)								
		−273.333	2615.410	−1377.90	0.968	0.937	0.926	44.30	88.78	0.0
	15	$\mathrm{Level}\;\mathrm{of}\;\mathrm{pressure}\;(2\;*)\text{:}\;E_{b}=b_{0}+b_{1}\cdot Ratio+b_{3}\cdot Ratio^{2}$ (5)								
		867.4667	1847.981	−1097.52	0.943	0.889	0.871	66.08	48.49	0.0
	15	$\mathrm{Level}\;\mathrm{of}\;\mathrm{pressure}\;(3\;*)\text{:}\;E_{b}=b_{0}+b_{1}\cdot Ratio+b_{3}\cdot Ratio^{2}$ (6)								
		2227.133	−945.829	323.0476	0.949	0.901	0.884	41.25	54.52	0.0
Tensile strength										
3c	15	$\mathrm{Level}\;\mathrm{of}\;\mathrm{pressure}\;(1\;*)\text{:}\;\sigma_{t}=b_{0}+b_{1}\cdot Ratio$ (7)								
		1.94780	2.19347		0.962	0.925	0.91	0.238	159.5	0.0
	15	$\mathrm{Level}\;\mathrm{of}\;\mathrm{pressure}\;(2\;*)\text{:}\;\sigma_{t}=b_{0}+b_{1}\cdot Ratio+b_{3}\cdot Ratio^{2}$ (8)								
		−0.92500	16.08992	−7.84343	0.935	0.873	0.852	0.354	41.43	0.0
	15	$\mathrm{Level}\;\mathrm{of}\;\mathrm{pressure}\;(3\;*)\text{:}\;\sigma_{b}=b_{0}+b_{1}\cdot Ratio+b_{3}\cdot Ratio^{2}$ (9)								
		0.800133	11.94787	−5.58133	0.919	0.844	0.818	0.311	32.49	0.0
Tensile modulus of elasticity										
3d	15	$\mathrm{Level}\;\mathrm{of}\;\mathrm{pressure}\;(1\;*)\text{:}\;E_{t}=b_{0}+b_{1}\cdot Ratio$ (10)								
		5.60200	9.98400		0.983	0.967	0.965	0.698	383.8	0.0
	15	$\mathrm{Level}\;\mathrm{of}\;\mathrm{pressure}\;(2\;*)\text{:}\;E_{t}=b_{0}+b_{1}\cdot Ratio+b_{3}\cdot Ratio^{2}$ (11)								
		10.93333	10.26667	−4.80000	0.923	0.851	0.826	0.259	34.31	0.00
	15	$\mathrm{Level}\;\mathrm{of}\;\mathrm{pressure}\;(3\;*)\text{:}\;E_{t}=b_{0}+b_{1}\cdot Ratio+b_{3}\cdot Ratio^{2}$ (12)								
		11.74000	10.80381	−5.02857	0914	0.835	0.808	0.292	30.45	0.00
Compressive stress at 10% deformation										
3e	15	$\mathrm{Level}\;\mathrm{of}\;\mathrm{pressure}\;(1\;*)\text{:}\;\sigma_{c}=b_{0}+b_{1}\cdot Ratio$ (13)								
		0.850093	4.98632		0.968	0.938	0.933	0.487	196.3	0.0
	15	$\mathrm{Level}\;\mathrm{of}\;\mathrm{pressure}\;(2\;*)\text{:}\;\sigma_{c}=b_{0}+b_{1}\cdot Ratio$ (14)								
		3.146200	5.46347		0.971	0.943	0.939	0.509	215.8	0.0
	15	$\mathrm{Level}\;\mathrm{of}\;\mathrm{pressure}\;(3\;*)\text{:}\;\sigma_{c}=b_{0}+b_{1}\cdot Ratio+b_{3}\cdot Ratio^{2}$ (15)								
		−0.129067	20.58166	−9.56076	0.925	0.856	0.832	0.516	35.63	0.0
Compressive modulus of elasticity										
3f	15	$\mathrm{Level}\;\mathrm{of}\;\mathrm{pressure}\;(1\;*)\text{:}\;E_{c}=b_{0}+b_{1}\cdot Ratio$ (16)								
		−0.563333	87.60800		0.961	0.923	0.917	9.616	155.6	0.0
	15	$\mathrm{Level}\;\mathrm{of}\;\mathrm{pressure}\;(2\;*)\text{:}\;E_{c}=b_{0}+b_{1}\cdot Ratio$ (17)								
		29.52000	84.82667		0.949	0.901	0.894	10.651	118.9	0.0
	15	$\mathrm{Level}\;\mathrm{of}\;\mathrm{pressure}\;(3\;*)\text{:}\;E_{c}=b_{0}+b_{1}\cdot Ratio$ (18)								
		49.68533	83.82400		0.953	0.908	0.901	10.110	128.9	0.0

* Note: Level of mixture pressure, in MPa: (1)—1.5; (2)—3.0; (3)—4.5 (Figure 3).

**Table 3:** Statistical analysis of the processing of engineered wood density results.

Figure	Specimen	Statistical Characteristics							
		$b_{0}$	$b_{1}$	R	R^2^	Adjusted R^2^	$S_{r}$	F	*p*
Density									
4	45	Level of pressure (1 *) $\rho =b_{0}+b_{1}\cdot Ratio$ (19)							
		290.3556	277.6444	0.980	0.961	0.960	20.36	1046.5	0.0
	45	Level of pressure (2 *) $\rho =b_{0}+b_{1}\cdot Ratio$ (20)							
		502.4444	201.1556	0.941	0.886	0.883	26.09	334.6	0.0
	45	Level of pressure (3 *) $\rho =b_{0}+b_{1}\cdot Ratio$ (21)							
		650.3778	119.6444	0.873	0.762	0.757	24.16	137.9	0.0

* Note: Level of mixture pressure, in MPa: (1)—1.5; (2)—3.0; (3)—4.5.

**Table 4:** Statistical analysis of the processing of engineered wood swelling results.

Figure	Specimen	Statistical Characteristics								
		$b_{0}$	$b_{1}$	$b_{2}$	R	R^2^	Adjusted R^2^	$S_{r}$	F	*p*
**G—swelling**										
5a	15	$\mathrm{Level}\;\mathrm{of}\;\mathrm{pressure}\;(1\;*)\;G=b_{0}+b_{1}\cdot Ratio+b_{3}\cdot Ratio^{2}$ (22)								
		16.32000	−9.17524	3.314286	0.942	0.887	0.868	0.386	46.88	0.0
	15	$\mathrm{Level}\;\mathrm{of}\;\mathrm{pressure}\;(2\;*)\;G=b_{0}+b_{1}\cdot Ratio+b_{3}\cdot Ratio^{2}$ (23)								
		41.85333	−52.0571	21.02857	0.987	0.974	0.969	0.763	223.3	0.0
	15	$\mathrm{Level}\;\mathrm{of}\;\mathrm{pressure}\;(3\;*)\;G=b_{0}+b_{1}\cdot Ratio+b_{3}\cdot Ratio^{2}$ (24)								
		50.16667	−62.1638	25.82857	0.963	0.928	0.916	1.431	77.28	0.0
**G—swelling**										
5b	18	$\mathrm{Level}\;\mathrm{of}\;\mathrm{pressure}\;(1\;*\;\mathrm{and}\;2\;*)\;\left(anova\right)$								
					0.680	0.463	0.239	0.679	2.067	0.14
	9	$\mathrm{Level}\;\mathrm{of}\;\mathrm{pressure}\;(3\;*)\;\left(anova\right)$								
					0.469	0.220	−0.0402	1.109	0.845	0.47

* Note: Level of mixture pressure, in MPa: (1)—1.5; (2)—3.0; (3)—4.5.

**Table 5:** Statistical analysis of the processing of engineered wood water absorption results.

Figure	Specimen	Statistical Characteristics								
		$b_{0}$	$b_{1}$	$b_{2}$	R	R^2^	Adjusted R^2^	$S_{r}$	F	*p*
**Water absorption**										
6a	15	$\mathrm{Level}\;\mathrm{of}\;\mathrm{pressure}\;(1\;*)\;W_{p}=b_{0}+b_{1}\cdot Ratio+b_{3}\cdot Ratio^{2}$ (25)								
		71.85961	−94.5193	37.21578	0.979	0.959	0.952	1.876	139.8	0.0
	15	$\mathrm{Level}\;\mathrm{of}\;\mathrm{pressure}\;(2\;*)\;W_{p}=b_{0}+b_{1}\cdot Ratio+b_{3}\cdot Ratio^{2}$ (26)								
		50.97333	−65.3257	26.20952	0.980	0.960	0.954	1.212	144.6	0.0
	15	$\mathrm{Level}\;\mathrm{of}\;\mathrm{pressure}\;(3\;*)\;W_{p}=b_{0}+b_{1}\cdot Ratio+b_{3}\cdot Ratio^{2}$ (27)								
		57.81333	−71.5600	29.06667	0.985	0.970	0.965	1.101	196.6	0.0
**Water absorption**										
6b	9	$\mathrm{Level}\;\mathrm{of}\;\mathrm{pressure}\;(1\;*)\;\left(anova\right)$								
					0.771	0.595	0.460	0.981	4.404	0.067
	9	$\mathrm{Level}\;\mathrm{of}\;\mathrm{pressure}\;(2\;*)\;\left(anova\right)$								
					0.427	0.183	−0.0897	0.644	0.671	0.54
	9	$\mathrm{Level}\;\mathrm{of}\;\mathrm{pressure}\;(3\;*)\;\left(anova\right)$								
					0.571	0.326	0.101	0.546	1.448	0.31

* Note: Level of pressure, in MPa: (1)—1.5; (2)—3.0; (3)—4.5.

**Table 6:** Analysis of physical properties.

Content of Expandable Graphite	Bending Strength, MPa		Tensile Strength, MPa		Compressive Stress, MPa		Swelling, %		Water Absorption, %	
	$\bar{x}$	$S_{r}$	$\bar{x}$	$S_{r}$	$\bar{x}$	$S_{r}$	$\bar{x}$	$S_{r}$	$\bar{x}$	$S_{r}$
0%	14.1	0.293	7.2	0.439	8.7	0.294	11.0	0.833	11.9	0.557
1%	14.3	0.259	7.1	0.141	8.9	0.105	10.7	0.321	11.8	0.280
3%	14.1	0.110	7.3	0.147	8.8	0.182	11.2	0.231	12.0	0.0971
5%	14.3	0.225	7.2	0.0378	8.9	0.170	10.9	0.351	11.9	0.263
Mean	14.20	0.227	7.21	0.224	8.84	0.197	10.97	0.452	11.93	0.300
Anova										
F(3,8)	0.832		0.450		0.920		0.411		0.163	
*p*(0.05)	0.513		0.724		0.474		0.750		0.918	
F_cr_	4.066									

**Table 7:** Thermal stability results of engineered wood composites at binder/filler ratio 1.00.

Amount of Expandable Graphite, wt.%	T, °C			Char Yield at 800 °C, wt.%
	T_5wt.%_	T_50wt.%_	T_max._	
0	219	356	346	20.4
1	247	356	344	22.5
3	231	356	341	23.7
5	217	356	341	25.8

## Data Availability

All data is contained within the article.
